# An FBG Optical Approach to Thermal Expansion Measurements under Hydrostatic Pressure

**DOI:** 10.3390/s17112543

**Published:** 2017-11-04

**Authors:** Priscila F. S. Rosa, Sean M. Thomas, Fedor F. Balakirev, Jon Betts, Soonbeom Seo, Eric D. Bauer, Joe D. Thompson, Marcelo Jaime

**Affiliations:** 1 Condensed Matter and Magnet Science Group, Los Alamos National Laboratory, MS K764, Los Alamos, NM 87545, USA; smthomas@lanl.gov (S.M.T.); soonbeom@lanl.gov (S.S.); edbauer@lanl.gov (E.D.B.); jdt@lanl.gov (J.D.T.); 2 National High Magnetic Field Laboratory, Los Alamos National Laboratory, MS E536, Los Alamos, NM 87545, USA; fedor@lanl.gov (F.F.B.); jbbetts@lanl.gov (J.B.); mjaime@lanl.gov (M.J.); 3 Institute for Materials Science, Los Alamos National Laboratory, Los Alamos, NM 87545, USA

**Keywords:** optical fiber Bragg grating, hydrostatic pressure, quantum criticality

## Abstract

We report on an optical technique for measuring thermal expansion and magnetostriction at cryogenic temperatures and under applied hydrostatic pressures of 2.0 GPa. Optical fiber Bragg gratings inside a clamp-type pressure chamber are used to measure the strain in a millimeter-sized sample of CeRhIn${}_{5}$. We describe the simultaneous measurement of two Bragg gratings in a single optical fiber using an optical sensing instrument capable of resolving changes in length $\left[dL/L=\left(L-L_{0}\right)/L_{0}\right]$ on the order of $10^{-7}$. Our results demonstrate the possibility of performing high-resolution thermal expansion measurements under hydrostatic pressure, a capability previously hindered by the small working volumes typical of pressure cells.

## 1. Introduction

The thermal expansion of a material provides important information to a broad range of fields. Different materials exhibit different changes in length in response to variations in temperature, making the study of thermal expansion crucial to the design of engines, bridges and space shuttles. The anomalous thermal expansion of water, for instance, has a strong impact on biological systems and needs to be accounted for in realistic simulations. In physics, thermal expansion measurements have been essential to determine the nature of critical phenomena (the peculiar behavior of a material when it is near a continuous phase transition) because the underlying crystal lattice is generally affected by criticality in either classical (T≠0) or quantum (T→0) phase transitions [1].

Unlike classical transitions, however, quantum fluctuations generated at a quantum critical point (QCP) dominate the physical properties of a material over large temperature ranges above T=0. This leads to anomalous thermodynamic behavior found experimentally in an increasing number of systems, heavy-fermion compounds being a prominent example. The theoretical understanding of quantum phase transitions, however, remains a major unsolved issue. Although there are a few proposed models, a controlled theory of a QCP is still lacking [2,3]. The electronic Grüneisen parameter, $\Gamma_{cr}$, is an important thermodynamic quantity to identify and classify quantum phase transitions because it diverges near a pressure-driven QCP with characteristic exponents for a given theory [4]. This parameter is defined as the ratio between the electronic contributions to the thermal expansion coefficient (α) and the specific heat ($c_{p}$), i.e., $\Gamma_{cr}\equiv \alpha /c_{p}=-\left(1/V_{m}T\right)\left(\partial S/\partial p\right)\left(\partial S/\partial T\right)$, where *S* is the entropy and $V_{m}$ is the molar volume.

Precise thermal expansion measurements under applied hydrostatic pressure, however, are impossible with a standard capacitance dilatometer technique. Capacitance dilatometry uses two capacitor plates, one fixed and one movable in contact with the sample, to measure the change in sample length, which manifests itself as a change in the gap between the capacitor pair [5,6]. The sensitivity of this technique can be very high ($dL/L∼10^{-8}$), but the dilatometer is sensitive to vibrations and electromagnetic interference, and the limited space inside a pressure cell (∼2 ×2×8 mm${}^{3}$) would substantially decrease the sensitivity of the measurement. Another electrical approach would be the use of resistive foil strain gauges, whose voltage output changes in response to variations in sample length. In this case, however, the resolution is considerably lower ($dL/L∼10^{-5}$) [7].

Here, we propose an optical approach to this experimental challenge by means of fiber Bragg grating (FBG) sensors. FBGs are fabricated using ultraviolet light to permanently change the index of refraction of the fiber core in a periodic manner [8]. This periodic modulation behaves as a selective mirror that only reflects a particular wavelength that satisfies the Bragg condition λ=2nΛ, where Λ is the grating pitch and *n* is the effective index of refraction of the core. By attaching a sample to the FBG, one can monitor the shift in wavelength of the reflected signal as a function of an external parameter (e.g., temperature, strain, pressure, magnetic field) and, consequently, the change in length of the sample [9]. More specifically, the wavelength shift is given by:

(1) $$\frac{\Delta \lambda }{\lambda }=\left\{1-\frac{n^{2}}{2}[P_{12}-\nu \left(P_{11}+P_{12}\right)]\right\}\frac{\Delta L}{L}+\left[\alpha +\frac{1}{n}\frac{dn}{dT}\right]\Delta T$$

where $P_{i,j}$ are the Pockel’s (piezo) coefficients of the stress-optic tensor, ν is the Poisson’s ratio, ΔL/L is the strain caused by the sample and α is the coefficient of thermal expansion of the fiber material. The photoelastic coefficient $\left(n^{2}/2\right)\left[P_{12}-\nu \left(P_{11}+P_{12}\right)\right]=0.22$ describes the sensitivity to strain in the 1550-nm band and is virtually temperature independent. The main temperature dependence of λ comes from the thermo-optic coefficient, ξ=(1/n)(dn/dT), which ranges from 8.3–9.5 × $10^{-6}/$K at room temperature. Although ξ is a non-linear function of *T* at high temperatures, it approaches zero as T→0 [10]. Moreover, λ is not expected to have magnetic field dependence when depolarized light is used to interrogate the FBG sensor [11]. We note that recent studies employ electroforming long-period fiber gratings as sensitive magnetic sensors [12].

In the following, we discuss the first implementation of thermal expansion and magnetostriction measurements using FBG sensors at cryogenic temperatures and hydrostatic pressures of 2.0 GPa. We note that FBG measurements under pressure have been reported, but only within the MPa range [13]. Our results reveal the advantages and the limitations of this technique in the GPa region and open an unexplored route to the understanding of materials under pressure.

## 2. Materials and Methods

**Figure 1:**
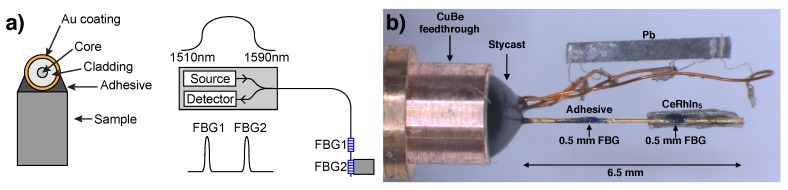
(**a**) (Left) Cross-section of our experimental setup. (Right) Schematics of the interrogation system. (**b**) Picture of the experimental setup for pressure-dependent measurements.

Low-cost optical fibers containing FBG sensors with λ∼1550 nm can be obtained commercially due to the advance of the fiber-optic communication industry over the past two decades. A typical fiber cross-section is shown in Figure 1a. The fiber core has a diameter of 9 μm, whereas the cladding reaches 125 μm. To prevent the fiber from breaking during manipulation, a protective coating is applied on top of the cladding. One of the most popular choices is polyimide owing to its good thermal properties and strong adhesion to the fiber, which yields accurate transmission of strain. Polyimide-coated fibers, however, cannot withstand high applied pressures and break at the point where it enters the pressure chamber. This issue can be solved by using a metal-coated fiber. Unlike in the polyimide coating, the axial stress in the metal coating is not negligible, and its high stiffness provides a higher load-carrying capacity in the axial direction. Further, recent strain transfer calculations indicate that accurate strain measurements can be performed with metal-coated FBGs as long as a thin metallic layer is used [14]. To interrogate the FBG sensors, we use a commercial swept wavelength laser that sweeps the wavelength between 1510 nm and 1590 nm at 5 kHz, as illustrated in Figure 1a [15]. To avoid birefringence effects due to the applied pressure, the laser outputs depolarized light. The low power of this laser (<0.25 mW) allows measurements in He${}^{3}$ temperatures. Measurements down to 2 K were performed in a Quantum Design PPMS system and measurements down to 400 mK were performed in an Oxford magnet equipped with a He${}^{3}$ insert. A temperature controller was used to vary the temperature at a rate of 0.1 K/min, ensuring thermalization of the pressure cell. Calibrated Cernox^®^ sensors (model CX) in thermal contact with the pressure cell were used as thermometers.

Figure 1b shows a picture of the experimental pressure setup. The gold-coated fiber and two copper twisted pairs are fed through a CuBe plug followed by the application of Stycast 2850 FT, which will hold the pressure in the pressure cell. We make use of the multiplexing capability of this technique to measure simultaneously two 0.5 mm-long FBG sensors (blue marks in Figure 1b) separated by 2 mm. Four 25-μm Pt wires were spot welded to a piece of Pb whose change in superconducting transition temperature (*T*${}_{c}$) served as a manometer. A Teflon cup, filled with Daphne oil 7373, is used to close the pressure chamber, which is then loaded in a hybrid piston-cylinder pressure cell.

An *a*-axis needle-like crystal of CeRhIn${}_{5}$ was bonded directly to the second FBG sensor ($\lambda_{2}=1545$ nm). Loctite^®^ 406 and Pattex^®^ ultra gel (cyanoacrylates) proved to be suitable adhesives. These adhesives cure quickly at room temperature and bond well to metals. We note that the Young’s moduli of silica and gold are ≈70–80 GPa, similar to the Young’s modulus of CeRhIn${}_{5}$. Therefore, the response of CeRhIn${}_{5}$ will dominate the response of the FBG as long as its cross-section is much larger than the (fiber + adhesive) cross-section [16].

**Figure 2:**
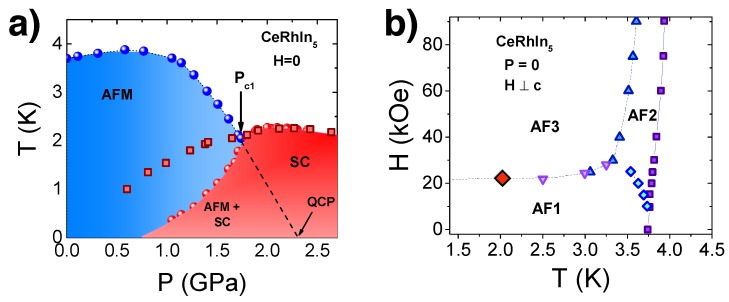
(**a**) T−P phase diagram of CeRhIn_5_ at zero magnetic field adapted from [21]; (**b**) field vs. temperature phase diagram of CeRhIn_5_ at zero pressure adapted from [23]. The diamond data point was obtained in this work.

Single crystalline samples of CeRhIn${}_{5}$ were grown by the In-flux technique. CeRhIn${}_{5}$ is tetragonal, and its crystallographic orientation was verified by an X-ray diffraction rocking curve at room temperature. CeRhIn${}_{5}$ is a highly tunable and exceptionally impurity-free antiferromagnetic metal and has become a prototype of quantum criticality. At atmospheric pressure, CeRhIn${}_{5}$ orders antiferromagnetically at $T_{N}=3.8$ K with a small ordered moment of 0.5 μB due to the Kondo effect [17,18,19]. Pressurizing CeRhIn${}_{5}$ tunes its magnetic transition toward a quantum-critical point, inducing unconventional superconductivity (USC) that coexists with the antiferromagnetic (AFM) order for pressures up to $P_{c1}=1.75$ GPa (Figure 2a). Above $P_{c1}$, evidence for $T_{N}$ is absent, and a dome of USC is observed centered over its pressure-tuned QCP [20,21,22]. At zero pressure, the field-temperature phase diagram of CeRhIn${}_{5}$ is displayed in Figure 2b when the field is applied in the ab-plane.

## 3. Results and Discussion

**Figure 3:**
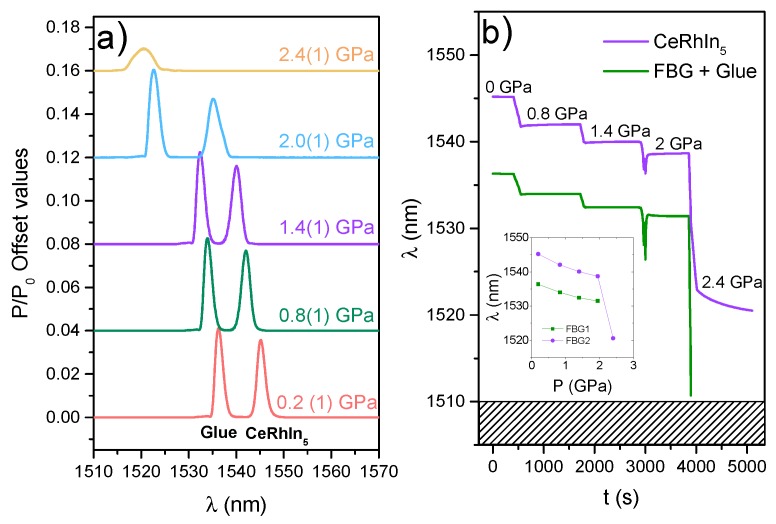
(**a**) Spectra of two FBG sensors at room-temperature as a function of applied pressure; (**b**) time evolution of the wavelength of the two FBGs as pressure is applied.

Figure 3a shows the spectra at room temperature of two FBG sensors as a function of applied pressure. At atmospheric pressure, the first FBG at ∼1535 nm contains only a small amount of adhesive, and the second FBG at ∼1545 nm contains the *a*-axis needle of CeRhIn${}_{5}$. As pressure is applied, both peaks shift to a lower wavelength, as expected from the compression of both the FBG and the sample under hydrostatic pressure.

Figure 3b shows the evolution of the peaks as a function of time as pressure is applied at room temperature in a hydraulic press. At low pressures, the change in wavelength is linear with applied pressure. At ∼2 GPa, however, the pressure medium solidifies at room temperature creating a less-hydrostatic environment. Although there is a spectral shape distortion due to this non-uniform pressure, it is still possible to obtain accurate data at ∼2 GPa. As pressure is further increased, however, the wavelength blue-shift responds more rapidly to applied pressure, and at ∼2.4 GPa, the first FBG peak shifts beyond the detection threshold (1510 nm) and cannot be observed anymore. Further, the spectral shape distortion becomes more pronounced, especially for the FBG containing the sample, and the peak detection becomes challenging.

**Figure 4:**
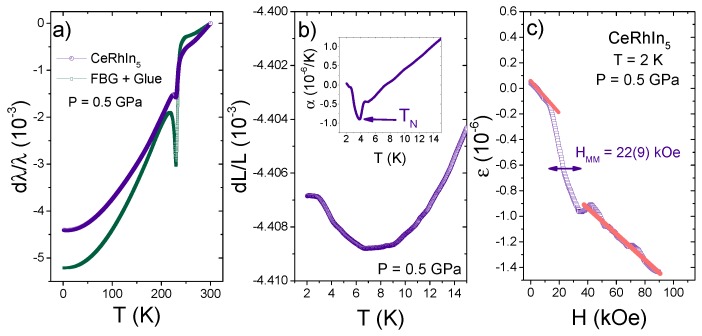
(**a**) Wavelength shift, Δλ/λ, as a function of temperature for two FBG sensors at 0.5 GPa: one containing a small amount of adhesive and the other one containing an a−axis needle of CeRhIn${}_{5}$. (**b**) Low temperature thermal expansion, ΔL/L, of CeRhIn${}_{5}$ at 0.5 GPa. The inset shows the *T*-dependence of the coefficient of thermal expansion. (**c**) Magnetostriction of CeRhIn${}_{5}$ at 2 K with the H||a-axis.

Figure 4 summarizes the properties of the FBG sensors at 0.5 GPa. Figure 4a shows the temperature dependence of the observed shift $d\lambda /\lambda =\left(\lambda -\lambda_{300\;K}\right)/\lambda_{300\;K}$, which includes both strain (ΔL/L) and thermal effects (ΔT) as shown in Equation (1). In principle, it is possible to compensate for thermal effects in the FBG containing the sample by measuring another FBG in close thermal contact with it, but completely unstrained. Therefore, by subtracting the wavelength of this FBG “temperature sensor” from the wavelength of the sample FBG, one would obtain the temperature-compensated strain value of CeRhIn${}_{5}$. This may be the case at temperatures above 250 K where $|\Delta \lambda_{CeRhIn_{5}}|>|\Delta \lambda_{Ref}|$, and consequently, CeRhIn${}_{5}$ contracts with decreasing temperature, as reported previously. At $T_{f}=230$ K, however, Daphne oil 7373 freezes and a clear anomaly is observed in both FBG sensors. Further, $|\Delta \lambda_{CeRhIn_{5}}|$ becomes smaller than $|\Delta \lambda_{Ref}|$, implying that CeRhIn${}_{5}$ displays an apparent (and incorrect) expansion with decreasing temperature. The fact that the bare FBG contracts more than the FBG with CeRhIn${}_{5}$ below $T_{f}$ indicates that the bare FBG is free to respond to compression from the solid pressure medium around it, whereas the second FBG is restrained by the response of the sample. The larger anomaly at $T_{f}$ observed in the bare fiber also supports this scenario. Therefore, we conclude that any thermal expansion measurement at high temperatures is hindered by the solidification of the pressure medium. We note, however, that $T_{f}$ gives a means of measuring the actual pressure inside the pressure cell. Daphne oil freezes at $T_{f}=176$ K at atmospheric pressure and at room-temperature at 2 GPa. Therefore, a simple linear extrapolation between these two points gives a pressure of 0.8 GPa at high temperatures. This is in agreement with the low-*T* measurement of Pb (P=0.5 GPa) because pressure decreases with decreasing temperature due to the differential contraction of the pressure cell parts. The associated ΔP is about 0.3 GPa at low pressures [24]. This sensitivity to the solidification of the medium along with the linear behavior of λ with pressure at a given temperature (Figure 3) reveals that FBG sensors can be used as pressure sensors in applications where hydrostatic pressure is examined.

It has been shown, however, that the temperature-dependent contribution to Δλ/λ approaches zero as T→0 [10], which makes Δλ/λ proportional to ΔL/L (Equation (1)). Therefore, we will focus our attention on the low-temperature region T<15 K from now on. Figure 4b shows ΔL/L vs. temperature of CeRhIn${}_{5}$ at 0.5 GPa. An upturn is clearly observed around 8 K, in agreement with previous measurements. Further, the first derivative of the data provides the coefficient of thermal expansion, $\alpha_{a}\left(T\right)$, shown in the inset of Figure 4b. Above 8 K, $\alpha_{a}\left(T\right)$ is positive, as expected from the contraction with decreasing temperature. Below 8 K, $\alpha_{a}\left(T\right)$ changes sign, and a peak appears at 3.9 K, signaling the second-order phase transition from a paramagnetic to an antiferromagnetic phase. This result is in qualitative agreement with the peak at $T_{N}$ seen by capacitance dilatometry at zero pressure [25,26] and with the phase diagram shown in Figure 2a where $T_{N}$ is 3.9 K at 0.5 GPa. The magnitude of the peak at $T_{N}$, however, is two- to three-times smaller than the value obtained using a capacitance dilatometer at atmospheric pressure. This reduction is likely a combination of two factors. First, there is an imperfect transmission of strain from the sample to the FBG sensor due to small FBG length, as well as the presence of the adhesive and the metallic coating layers. In an FBG measurement, it is known that part of the strain is transferred to shear stress on the intermediate layers [16]. Secondly, the slope $dT_{N}/dP$ at 0.5 GPa (Figure 2a) is notably less than at lower pressures. By Ehrenfest’s relation for a second order phase transition, the weaker slope necessarily implies a smaller thermal expansion anomaly at $T_{N}$, if the change in specific heat across the phase boundary is similar to that at atmospheric pressure, which it is [25,26]. Hence, the temperature dependence of α(T) is reliable although its absolute value may not be quantitative. Figure 4c shows the magnetostriction of CeRhIn${}_{5}$ at 2 K. The magnetostriction is negative, as reported previously, and shows a broad transition centered at ∼22 kOe, in agreement with the reported H−T phase diagram (diamond data point shown in Figure 2b). Previous specific heat measurements revealed that the zero-pressure H−T phase diagram does not change significantly at low applied pressures [21].

**Figure 5:**
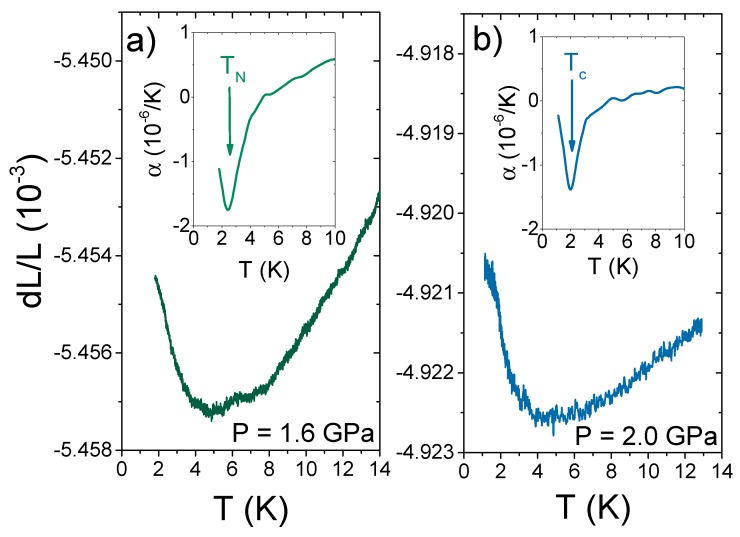
(**a**) Low temperature thermal expansion, $\Delta L_{a}/L_{a}$, of CeRhIn${}_{5}$ at 1.6 GPa; (**b**) low temperature thermal expansion, $\Delta L_{a}/L_{a}$, of CeRhIn${}_{5}$ at 2.0 GPa. Insets show the temperature dependence of the coefficient of thermal expansion.

Figure 5a shows the low-temperature dL/L raw data at 1.6 GPa, just below $P_{c1}=1.75$ GPa. Both the upturn and the kink at $T_{N}$ shift to lower temperatures. In the inset, $\alpha_{a}\left(T\right)$ displays a minimum at $T_{N}=2.4$ K, in agreement with the phase diagram shown in Figure 2a. The magnitude of this minimum, however, is almost two-times larger than the value at 0.5 GPa. According to Ehrenfest’s relation, the pressure derivative of $T_{N}$ can be written as $dT_{N}/dP=2V_{m}\left[\Delta \alpha_{a,b}/\left(\Delta C_{p}/T_{N}\right)\right]+V_{m}\left[\Delta \alpha_{c}/\left(\Delta C_{p}/T_{N}\right)\right]$ in a tetragonal material such as CeRhIn${}_{5}$ [27]. Here, Δα and $\Delta C_{p}$ are the discontinuities of the linear thermal-expansion coefficients and the specific heat at $T_{N}$, respectively. Because the jump in heat capacity at $T_{N}$ is always positive, the signs and relative magnitudes of the pressure derivative components are given directly by the thermal expansion jumps. At about 0.5 GPa, $T_{N}$ goes through a maximum, which gives $dT_{N}/dP\approx 0$. This implies that $\Delta \alpha_{c}$ will be opposite in sign and its magnitude will equal $2\Delta \alpha_{a}$. As a matter of fact, $\Delta \alpha_{c}$ is positive at atmospheric pressure with a magnitude four-times larger than $\Delta \alpha_{a}$, and we expect this magnitude to decrease under pressure to compensate $\Delta \alpha_{a}$. At higher pressures, $T_{N}$ starts to decrease at a rate of ≈−3.2 K/GPa, which is likely the reason for the increase in the $\Delta \alpha_{a}$ minimum at 1.6 GPa when compared to 0.5 GPa. Thermal expansion measurements along the *c*-axis are underway to confirm this scenario.

Figure 5b shows the low-temperature dL/L raw data at P=2.0 GPa, above $P_{c1}=1.75$ GPa. A kink is observed at about 2 K, which corresponds to the superconducting transition temperature, $T_{c}$, in the phase diagram shown in Figure 2b. In the inset, $\alpha_{a}\left(T\right)$ displays a minimum at $T_{c}=2$ K and tends to zero as T→0 K. This result is different from the thermal expansion of CeCoIn${}_{5}$, a similar *d*-wave superconductor already at ambient pressure. In CeCoIn${}_{5}$, $dL_{a}/L_{a}$ dominates the response of $\alpha_{V}$ and does not exhibit a minimum, yielding a positive α(T) and a maximum at $T_{c}$. Thermal expansion measurements along the *c*-axis are valuable to understand this difference because $dL_{c}/L_{c}$ may play a dominant role in determining the volumetric thermal expansion.

Finally, we note that metal-coated FBG sensors are at the forefront of the current technology and Au-coated fibers with higher quality are expected to be developed. Recent simulations of the strain transfer have shown the substantial effects of the transition layers and of the Bragg grating size. Therefore, FBGs with longer gratings could be used to improve sensitivity.

## 4. Conclusions

In summary, we describe an optical technique for measuring thermal expansion and magnetostriction at cryogenic temperatures (T<15 K) and under applied pressures of 2.0 GPa. Optical fiber Bragg gratings are used as strain sensors inside a conventional piston-cylinder-type pressure cell. Temperature compensation at high temperatures is hindered by the freezing point of Daphne oil 7373, the pressure transmitting medium. At low temperatures, however, temperature effects are negligible, and we were able to simultaneously measure two Bragg gratings using an optical sensing instrument capable of resolving strains in the order of $10^{-7}$. We demonstrate this optical approach in an *a*-axis sample of CeRhIn${}_{5}$, a heavy-fermion superconductor under applied pressure. Our results show the possibility of performing high-resolution thermal expansion measurements under pressure and open new possibilities for a broad range of materials.
